# Kinesiophobia Affect Postural Control Strategies During Jump Landing in Athletes With Chronic Ankle Instability: A Cross‐Sectional Study

**DOI:** 10.1002/hsr2.72266

**Published:** 2026-05-03

**Authors:** Sarah Jafari, Sedigheh Sadat Naimi, Zahra Ebrahimabadi, Marzieh Mortezanejad, Reihaneh Aakary kachoosangy, Sanaz Shanbehzadeh

**Affiliations:** ^1^ School of Rehabilitation, Physiotherapy Research center Shahid Beheshti University of Medical Sciences Tehran Iran; ^2^ Neuromuscular Rehabilitation Research Center, Research Institute of Nuerosciences Semnan University of Medical Sciences Semnan Iran; ^3^ Iranian Center of Excellence in Physiotherapy, Rehabilitation Research Center, Department of Physiotherapy, School of Rehabilitation Sciences Iran University of Medical Sciences Tehran Iran

**Keywords:** avoidance behaviour, chronic ankle instability, dynamic stability, fear, jumping, postural control

## Abstract

**Background and Aims:**

Altered postural control has been observed in chronic ankle instability (CAI). Kinesiophobia (fear of re‐injury) has been identified as a factor influencing postural stability. Therefore, postural control was assessed during jump landing among CAI athletes with and without kinesiophobia, as well as healthy controls.

**Methods:**

A total of 60 participants were recruited, including 40 individuals with CAI and 20 healthy controls. The CAI group was subdivided into two cohorts: 20 subjects with kinesiophobia (Tampa Scale of Kinesiophobia score > 37) and 20 without kinesiophobia. All participants performed a single‐leg jump landing task on a force plate. The following postural stability metrics were calculated: time to stabilization (TTS), and the mean and standard deviation of center of pressure (COP) displacement and velocity in the anterior–posterior (AP), medial–lateral (ML), and total directions.

**Results:**

CAI subjects with kinesiophobia exhibited longer TTSs in the AP (*p* = 0.019), vertical, and total directions (*p* < 0.001, *p* = 0.015, *p* = 0.002) than healthy subjects. Additionally, CAI subjects with kinesiophobia demonstrated a significant increase in the velocity of COP displacement in the AP and ML directions (*p* < 0.001, *p* = 0.004). These individuals showed greater variability in the velocity of COP displacement in the AP direction and in COP displacement in the ML direction than healthy subjects (*p* < 0.001). Also, CAI with kinesiophobia group presented less variability in the velocity of COP displacement in the ML direction than other groups (*p* < 0.001, *p* = 0.026). CAI with kinesiophobia group needed more time and more velocity in the AP direction than without kinesiophobia group (*p* = 0.003).

**Conclusion:**

Individuals with a high fear of re‐injury may perceive the jump landing task as threatening, requiring more time to land and regain stability. This fear can restrict the variability of velocity in the ML direction, resulting in decreased control over the AP direction due to limitations on managing degrees of freedom.

**Abbreviations**
ADL: Activities of Daily Living
AP: Anterior‐Posterior
AP‐TTS: Anterior‐Posterior time to stability
CAI: Chronic ankle instability
COP: Center of Pressure
d‐ML‐SD: Distance medial–lateral standard deviation
D–AP–SD: Distance anterior–posterior standard deviation
FAAM: Foot and Ankle Ability Measure
GRF: Ground reaction force
LAS: Lateral Ankle Sprain
ML: Medial–Lateral
SD: Standard Deviation
TSK: Tampa Scale of Kinesiophobia
TTS: Time to Stabilization
V–AP–SD: Velocity anterior–posterior standard deviation
V‐ML–SD: Velocity medial–lateral standard deviation
V‐TTS: Vertical time to stabilization
η_*p*_^2^
: Eta squared values

## 1 Introduction

Chronic ankle instability (CAI) is defined by recurring lateral ankle sprains (LAS), functional limitations, persistent pain, swelling, and frequent “giving way” episodes that continue for at least 12 months after the initial injury [1]. This condition is prevalent among athletes and is recognized as a typical musculoskeletal injury in sports contexts [2]. Such injuries can immediately hinder mobility and athletic involvement activities [3], with consequences that go beyond physical limitations to also affect psychological and social well‐being [4]. Athletes often fear reinjury, inability to return to peak performance, or the possibility of long‐term symptoms.

Individuals with CAI often report frequent episodes of instability, which can contribute to reduced physical activity and suboptimal athletic performance [1]. Research indicates that CAI results from a combination of mechanical, sensorimotor, and behavioral deficits [1]. Psychological elements such as fear of reinjury fall within the sensory‐cognitive category, while motor behavior plays a critical role in maintaining balance [1]. Evidence suggests that CAI patients commonly exhibit impaired postural control [5, 6, 7, 8, 9]. which is essential for executing athletic movements, particularly in complex and dynamic scenarios [10].

Neuroscientific studies reveal that brain regions involved in emotion and those governing postural regulation are interconnected [11, 12]. The limbic system and the amygdala—areas associated with emotional processing, including fear—project to motor areas involved in postural control [12]. As a result, emotional factors like fear may heighten the risk of postural control dysfunction [13]. Kinesiophobia, defined as an excessive and irrational fear of movement or physical activity stemming from fear of injury or reinjury, can be a significant barrier for functional recovery. This fear can negatively influence athletes' physical performance by decreasing muscle strength, impairing cognitive and motor function, and reducing range of motion [14, 15]. Consequently, individuals experiencing kinesiophobia may demonstrate increased vulnerability and poorer balance control [16].

Several investigations have shown a negative association between kinesiophobia and postural control in CAI patients during dynamic tasks [17, 18, 19]. A recent systematic review concluded that kinesiophobia can lead to weaker muscles, reduced proprioceptive accuracy, and limited joint mobility, all of which negatively affect athletic output [20]. Additional studies confirm the detrimental impact of psychological distress, particularly kinesiophobia, on both balance and physical function in CAI patients [17, 19, 21].

The existing literature consistently shows that kinesiophobia can hinder physical function and balance among athletes [17, 19, 21]. kinesiophobia have been linked with deteriorated postural stability [22], while individuals without kinesiophobia are associated with improved dynamic balance after ACL reconstruction [23], Conversely, individuals with greater fear of movement often adopt protective strategies that compromise postural control and physical activity. In people with musculoskeletal issues, including CAI, fear of reinjury may reduce activity levels and impair postural stability [24, 25]. Despite substantial evidence, the specific effect of kinesiophobia on balance in CAI has not yet been explored.

To evaluate postural control, dynamic tests such as the single‐leg vertical jump (SVJ) are utilized, which simulate real‐life athletic demands by requiring balance during the shift from motion to stillness. Using a force platform, researchers assess balance by examining center of pressure (COP) movement and ground reaction forces (GRF). COP displacement provides insights into both spatial and temporal balance characteristics and is a standard method for measuring static balance. Compared to traditional measures, time to stabilization (TTS) is a dynamic metric that may offer a more functional representation of postural performance. TTS measures how long it takes for the body to regain stability following landing and serves as an indicator of neuromuscular function and proprioceptive ability in the lower limbs [26].

Although prior research confirms that CAI patients with kinesiophobia exhibit balance impairments during functional tasks and static balance task [16, 17, 19, 21], but the impact of kinesiophobia on dynamic postural control remains unclear. Therefore, this study aimed to compare static and dynamic postural stability among CAI patients with and without kinesiophobia and healthy individuals. It was hypothesized that those with kinesiophobia would display greater COP displacement and longer TTS compared to other groups.

## 2 Methods

### 2.1 Study Design

This cross‐sectional research was carried out in the physiotherapy department of the ***** laboratory and received approval from the Human Research Ethics Committee at ***** (approval number: *****).

### 2.2 Participants

A total of 60 individuals participated in the study: 20 participants with CAI and kinesiophobia, 20 with CAI and without kinesiophobia—classified using a TSK score cutoff of ≤ 37 for without kinesiophobia and > 37 for accompanied with kinesiophobia [16]) and 20 healthy controls with no history of lateral ankle sprain.

Eligibility criteria included being a nonprofessional athlete engaged in over 6 h per week of club‐level sports involving jumping and directional changes, categorized as level I (e.g., basketball, handball, football) or level II (e.g., volleyball, racquet sports, martial arts, gymnastics) [27]. Participants must have experienced at least one unilateral ankle sprain over a year prior to the study that required protected weight‐bearing or immobilization for a minimum of 3 days [28]. Additionally, CAI subjects needed to have Foot and Ankle Ability Measure (FAAM) scores below 90% for daily living and below 80% for sports subscales [28]. Exclusion criteria comprised ongoing ankle pain or swelling, bilateral ankle sprains, visual or balance impairments, any condition affecting balance, lower limb surgeries or fractures, neurological disorders, and ankle pain or swelling within the past 3 months [28]. All participants signed a written informed consent form prior to the start of the study.

### 2.3 Outcome Measures Questionnaires

The FAAM and TSK questionnaires were completed by CAI athletes. The healthy controls completed only the TSK questionnaire. The Persian version of the TSK has shown good reliability (Cronbach's α ranging from 0.77 to 0.78) and validity [29]. The Persian version of the FAAM is a reliable and valid measure for quantifying physical functioning in patients with foot and ankle disorders [30]. The FAAM is a 29‐item questionnaire split into two subscales: the Daily Living Activities (ADL), with 21 items, and the SPORTS, with eight items. Each item is rated on a 5‐point Likert scale representing various levels of difficulty (no difficulty at all, slight difficulty, moderate difficulty, extreme difficulty, and inability to do so). The ADL and SPORTS subscales have overall scores of 84 and 32, respectively. Scores are converted into percentages, with higher scores indicating a higher functional status for each subscale. There are two scales for each FAAM subscale that require subjects to rate their current function during activities of daily living (ADL) and sports tasks on a scale from 0% to 100%. A rating of 0% indicates an inability to perform any task, whereas a rating of 100% indicates the performance before the injury. Another scale asks subjects to assess their current global function, with responses classified as normal, near normal, abnormal, or severely abnormal [30].

The TSK is a 17‐item instrument designed to measure pain‐related fear of new injury/motion. All the items are graded on a 4‐point [1, 2, 3, 4] Likert scale from strongly disagree to strongly agree. The TSK score ranges between 17 and 80, with higher scores indicating a greater degree of fear associated with motion/pain reidentification. The cutoff point for the Tampa Scale of Kinesiophobia was 37 [16].

### 2.4 COP Measures

Postural stability was assessed using a Bertec force plate (Bertec Corporation®, Columbus, USA) sampling at 1000 Hz. The study focused on COP displacement and velocity in the anterior‐posterior (AP) and mediolateral (ML) directions (COP x and y), as well as time to stabilization (TTS) in the AP, vertical, and combined directions. These indicators are commonly used to evaluate the spatial and temporal features of postural stability [31].

### 2.5 Procedures

**Figure 1:**
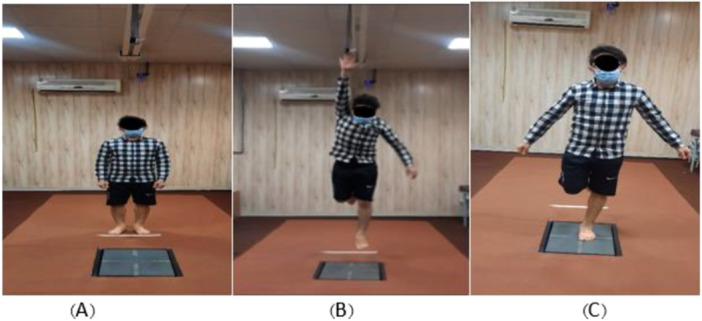
The steps of single leg jumping. The figure on the left shows the start of the test, while the figure on the right shows the subject completing the task. In Figure A, the athlete is standing with his lower limbs positioned halfway between the force plate and a white marker on the floor. (B) The athlete single jumped at the 50% of maximum height jump and (C) post landing time.

Participants began by standing behind the force plate at a distance equal to half their leg length. All trials were performed eyes‐open. Participants were instructed to maintain gaze on a fixed visual target positioned at eye level approximately 2–3 m in front of the force plate during landing and throughout the 10‐s stabilization period. Their maximum vertical jump height was determined before the trials. Each participant performed three bilateral jumps landing at 50% of their maximum vertical jump height (Figure 1) [31, 32]. They were allowed to use arm swings, touch targets, and then land on the affected ankle (or matched limb in controls). A familiarization session was conducted before testing. A valid trial required the participant to land on the force plate and maintain balance for at least 10 s post‐landing [33]. Each test was repeated three times, with 30‐second rest intervals. Trials were repeated or discarded if the participant landed incorrectly, left the platform, performed an unintended jump, or used excessive body movements [34]. Trials were repeated if participants did not comply with the visual instructions.

### 2.6 Dynamic Postural Control Parameters

#### 2.6.1 Measured Variables Included

- Total time to stabilization (Total‐TTS).
- Vertical time to stabilization (V‐TTS).
- Anterior‐posterior time to stabilization (AP‐TTS).
- Mean and standard deviation of COP velocity in AP (V‐AP‐Mean, V‐AP‐SD) and ML directions (V‐ML‐Mean, V‐ML‐SD).
- Mean and standard deviation of COP displacement in AP (d‐AP‐Mean, d‐AP‐SD) and ML directions (d‐ML‐Mean, d‐ML‐SD).

All AP variables were normalized to foot length, and ML variables to foot width to account for individual differences in body dimensions.

### 2.7 Data Processing

**Figure 2:**
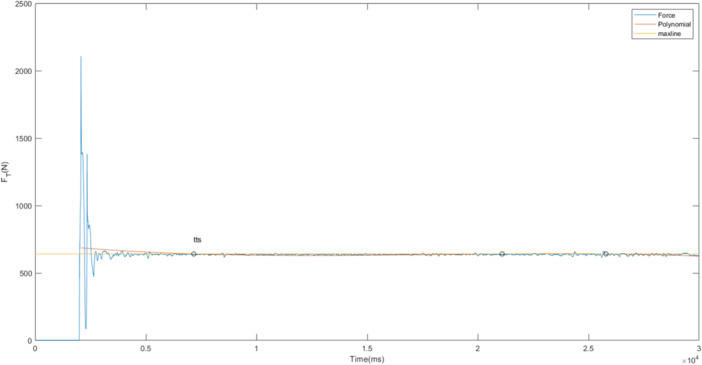
Time to stability in the medial‐lateral, anterior‐posterior, and vertical direction during jump landing.

**Figure 3:**
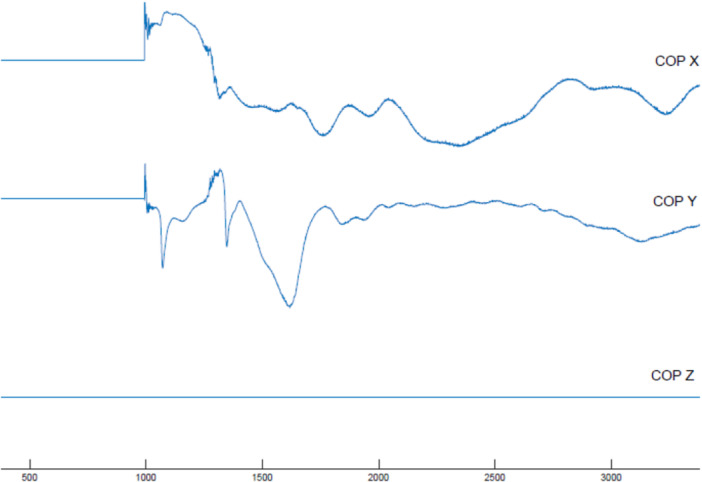
Center of pressure trace in the medial‐lateral, anterior‐posterior, and vertical direction during jump landing.

The analysis was based on the average of three single‐leg jump‐landing trials. Force plate data were collected at a sampling rate of 1000 Hz. Raw data were filtered using a bi‐directional 4th‐order Butterworth low‐pass filter with a cutoff frequency of 10 Hz. Data processing was performed using MATLAB R2018 (Mathworks™ Inc., Natick, MA, USA). The primary variables analyzed included time to stability (TTS) in the vertical, anterior‐posterior (AP), and combined directions (Figure 2). TTS was calculated using vertical ground reaction force (GRF), AP force, and a total force vector (a combination of forces in all three spatial directions). For instance, in the total force vector graph, the blue curve represents the force applied during a jump‐landing task. During landing, this force temporarily exceeds body weight due to impact but eventually stabilizes. To identify the TTS, the 30‐second trial was divided into 500 ms windows. Within each window, the range of force fluctuation (max ‐ min) was calculated. The window with the smallest force fluctuation was selected, and a horizontal reference line (Max Line) was drawn at the maximum force within that window. A polynomial curve was then fitted from the maximum force point to the end of the trial. The point where this curve first intersected the Max Line was defined as the TTS(Figure 2) [35]. Secondary outcomes included center of pressure (COP) metrics—specifically the peak‐to‐peak standard deviation and mean velocity of COP displacement in both the ML and AP directions—calculated over a 10‐second period after landing (Figure 3).

### 2.8 Statistical Analysis

SPSS version 27 was used for statistical evaluation. The Kolmogorov–Smirnov test checked for normal distribution, and Levene's test was used to assess homogeneity of variance. Depending on these results, either one‐way ANOVA or the Kruskal–Wallis test was applied. For post hoc analysis, Tukey's test was used when variance was equal; otherwise, the Games‐Howell test was applied. A significance α = 0.05 was used. The eta squared (η²) effect size was used for effect size calculation. Based on the η^2^ = 0.01 indicates a small effect, η^2^ = 0.06 indicates a medium effect, andη^2^ = 0.14 indicates a large effect [36].

### 2.9 Sample Size

Sample size estimation was conducted using the G*Power software (version 3.1.9.4) and pilot data from seven athletes per group, focusing on TTS during jump‐landing. Based on an effect size of 0.82, 90% statistical power, and α = 0.05, the minimum required sample size was 23 participants. Accounting for an anticipated dropout rate of 26%, a total of 60 participants (20 per group) were recruited.

## 3 Results

Table 1 presents the demographic data. Descriptive data about baseline variables are depicted in Table 2.

**Table 1:** The frequency distribution of gender and the mean and standard deviation of the background variables in each group.

Variable group	Healthy group	Without kinesiophobia CAI	With kinesiophobia CAI	*p* value
Age (years)^a^	23.50 ± 3.12	22.45 ± 4.14	24.70 ± 5.12	0.247
Height (m)^a^	1.78 ± 0.11	1.80 ± 0.13	1.71 ± 0.07	0.067
Weight (kg)^a^	71.30 ± 6.67	74.18 ± 9.99	67.70 ± 7.09	0.056
BMI (kg/m^2^)^a^	22.24 ± 1.45	22.80 ± 1.32	23.02 ± 1.39	0.195
Gender^b^ *N* (female/male)	7/13	8/12	7/13	
TSK score	31.65 ± 5.09	29.40 ± 6.80	49.80 ± 7.23	0.001
FAAM ADL^a^	—	45.80 ± 15.12	40.15.0 ± 15.13	0.275
FAAM SPORT^a^	—	67.15 ± 12.09	63.05 ± 14.36	0.335

*Note:* Data is presented as mean ± SD and frequency distribution.

Abbreviations: ADL, activity daily living; BMI, body mass index; CAI, chronic ankle instability; FAAM, foot and ankle measure; N, number; SD, standard deviation.

^a^ ANOVA test.

^b^ Chi square tests for demographic data.

**Table 2:** Descriptive data about baseline variables.

Variables	Group	Mean ± SD	Median ± variance	Minimum‐ maximum
Total_Tts (Second)^b^	CAI with kinesiophobia		3.22 ± 0.69	2.29–5.81
	CAI without kinesiophobia		2.93 ± 0.63	1.04–5.08
	Healthy controls		2.59 ± 0.81	1.40–4.99
Vertical_Tts (Second)^b^	CAI with kinesiophobia		3.22 ± 0.822	1.81–5.81
	CAI without kinesiophobia		2.90 ± 0.69	0.81–4.77
	Healthy controls		2.58 ± 0.95	1.19–5.09
AP_TTS (Second)^a^	CAI with kinesiophobia	0.026 ± 0.002		
	CAI without kinesiophobia	0.015 ± 0.001		
	Healthy controls	0.01 ± 0.002		
COP_ D_AP_MEAN (mm)^b^	CAI with kinesiophobia		0.05 ± 0.00	0.03–0.08
	CAI without kinesiophobia		0.044 ± 0.00	0.04–0.09
	Healthy controls		0.‐44 ± 0.001	0.03–0.20
COP_ D_AP_SD (mm)^b^	CAI with kinesiophobia		12.44 ± 9.64	8.32–18.88
	CAI without kinesiophobia		11.05 ± 12.41	8.41–22.64
	Healthy controls		10.59 ± 8.70	7.42–18.47
COP_ D‐ ML_MEAN (mm)^a^	CAI with kinesiophobia	39.266 ± 30.66		9.60–77.98
	CAI without kinesiophobia	36.05 ± 26.99		9.03–78.87
	Healthy controls	39.61 ± 30.05		10.41–78.24
COP_D_ML_SD (mm)^b^	CAI with kinesiophobia		0.137 ± 0.002	0.097–0.23
	CAI without kinesiophobia		0.122 ± 0.002	0.089–0.29
	Healthy controls		0.104 ± 0.001	0.067–0.19
COP _V_AP_MEAN (mm/S)^b^	CAI with kinesiophobia		0.32 ± 0.014	0.21–0.71
	CAI without kinesiophobia		0.25 ± 0.007	0.14–0.43
	Healthy controls		0.25 ± 0.01	0.16–0.59
COP _V_AP_SD (mm/S)^b^	CAI with kinesiophobia		0.25 ± 0.01	0.06–0.69
	CAI without kinesiophobia		0.21 ± 0.007	0.14–0.43
	Healthy controls		0.13 ± 0.01	0.06–0.69
COP_ V_ML_MEAN (mm/S)^b^	CAI with kinesiophobia		0.90 ± 0.05	0.45–1.43
	CAI without kinesiophobia		0.82 ± 0.05	0.61–1.35
	Healthy controls		0.61 ± 0.06	0.28–1.37
COP_ V_ML_SD (mm/S)^b^	CAI with kinesiophobia		0.42 ± 0.02	0.10–0.77
	CAI without kinesiophobia		0.57 ± 0.03	0.31–1.07
	Healthy controls		0.54 ± 0.03	0.307–1.15

*Note:* Data is reported as mean ± SD for normal distribution and median ± variance for abnormal distribution.

^a^ Normal data.

^b^ Abnormal data.

Neither ANOVA nor the Kruskal–Wallis test showed significant differences among the groups in demographic variables or FAAM scores (*p* > 0.056). However, significant group differences were observed in TTS in all directions (vertical, AP, and total) (*p* < 0.034). Additionally, there were significant differences in mean COP velocity in both the AP and ML directions (V_AP_Mean, V_ML_Mean), as well as in the variability of COP displacement and velocity in both planes (V_ML_SD, D_ML_SD, V_AP_SD) (Table 3).

**Table 3:** ANOVA results of dependent variables between three groups.

ANOVA				
Variables		*F*	Eta squard	*p*‐value
Total_Tts (Second)^b^	Between Groups	4.269	0.130	**0.006
Vertical_Tts (Second)^b^	Between Groups	2.984	0.095	**0.034
AP_TTS (Second)^a^	Between Groups	136.501	0.827	**0.000
COP D_AP_MEAN(mm)^b^	Between Groups	2.321	0.075	0.152
COP D_AP_SD(mm)^b^	Between Groups	1.433	0.048	0.160
COP D‐ ML_MEAN^a^ (mm)^b^	Between Groups	.204	0.07	0.816
COP D_ML_SD(mm)^b^	Between Groups	5.528	0.162	**0.006
COP V_AP_MEAN(mm/S)^b^	Between Groups	5.248	0.155	**0.008
COP V_AP_SD(mm/S)^b^	Between Groups	6.945	0.196	**0.002
COP V_ML_MEAN(mm/S)^b^	Between Groups	5.038	0.150	**0.010
COP V_ML_SD(mm/S)^b^	Between Groups	7.294	0.204	**0.002

*Not*e*:* ***p‐value < 0.001.

Abbreviations: AP, anterior–posterior; D, Distance; Mean, Average; ML, medial–lateral; mm, millimeter; SD, standard deviation; TTS, Time to stability; V, velocity.

* *p*‐value< 0.05

** *p*‐value < 0.01.

^a^ ANOVA.

^b^ Kruskal wallis tests for dependent variables between three groups.

### 3.1 Post Hoc Comparisons

- CAI with kinesiophobia versus Healthy Controls

**Figure 4:**
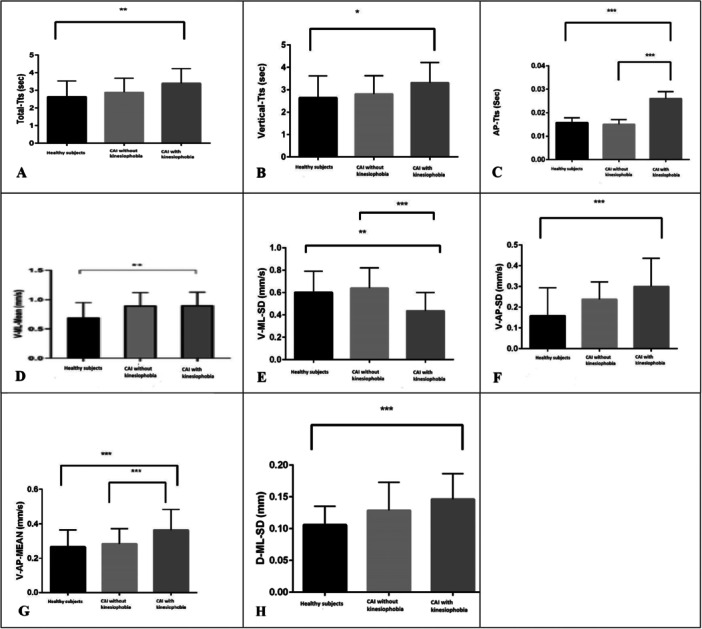
Paiwise comparison for the COP and TTS parameters between three group. (A) Time to stability in total direction, (B) time to stability in vertical direction, (C) time to stability in AP direction, (D) mean velocity in the ML direction, (E) Standard deviation of velocity in the ML direction, (F) Standard deviation of velocity in the AP direction, (G) mean Velocity in the AP direction, and (H) standard deviation of distance in the ML direction. *p‐value < 0.05. **p‐value < 0.01. ***p‐value < 0.001. AP, anterior‐posterior; COP, Center of pressure; D, distance; Mean, average; ML, medial‐lateral; SD, standard deviation; TTS, time to stabilization; V, velocity.

Participants with kinesiophobia showed significantly longer TTS in the vertical, AP, and total directions compared to healthy individuals (*p* < 0.001, *p* = 0.015, and *p* = 0.002, respectively), indicating delayed postural recovery after landing. They also had higher COP displacement velocities in both AP and ML directions (*p* < 0.001 and *p* = 0.004), greater variability in AP velocity and ML displacement of COP (both *p* < 0.001), but reduced variability in velocity of COP displacement in the ML direction (*p* = 0.003) (Figure 4).

- CAI With kinesiophobia versus CAI without Kinesiophobia

Compared to those without kinesiophobia, the CAI with kinesiophobia group exhibited longer TTS in the AP direction, and greater velocity of COP displacement in the AP direction after landing (*p* < 0.001 and *p* = 0.026). Additionally, they showed lower variability in the velocity of COP displacement in the ML direction than those without kinesiophobia (*p* < 0.001) (Figure 4).

## 4 Discussion

The purpose of this study was to investigate the role of kinesiophobia in postural control and landing stability among athletes with CAI using force‐plate–derived COP measures and TTS during a jump‐landing task. The main findings were that athletes with CAI and kinesiophobia demonstrated longer AP TTS and higher AP COP velocity compared with both CAI athletes without kinesiophobia and healthy controls. In contrast, ML COP velocity variability was reduced in the CAI with kinesiophobia group. These findings suggest that kinesiophobia is associated with measurable alterations in dynamic postural stabilization during landing, beyond the effects of CAI alone.

### 4.1 Differences Between CAI With Kinesiophobia and Healthy Subjects

Participants with CAI with kinesiophobia required significantly more time to regain balance across vertical, AP, and combined dimensions compared to healthy individuals. This delay may stem from a more cautious landing strategy intended to prevent further injury [37]. Our findings support the notion that CAI is associated with compromised landing stabilization, particularly in the sagittal plane, which plays a key role in shock absorption and forward momentum control during jump‐landing tasks [38].

Consistent with previous research, athletes with CAI exhibited altered postural control during dynamic tasks compared with healthy individuals [17, 18, 19]. Landing from a jump places high mechanical and sensorimotor demands on the ankle joint, requiring rapid force attenuation and efficient stabilization [16]. Prolonged TTS and altered COP behavior have been interpreted as indicators of reduced dynamic stability and delayed neuromuscular control following ground contact. TTS in the AP and total direction has shown a large effect size, while the vertical TTS showed a medium effect size. This suggested that the observed group differences in the vertical TTS parameter may have limited practical impact, whereas the other parameters showed a large practical effect.

### 4.2 Differences Between CAI With and Without Kinesiophobia

Individuals with kinesiophobia exhibited increased TTS in the AP direction, higher COP velocity in the AP direction, and reduced variability in ML displacement compared to those without kinesiophobia. The reduction in postural control in the AP direction might be a compensatory strategy to shield the ML structures typically involved in ankle sprains.

A key contribution of this study is the direct comparison between CAI athletes with and without kinesiophobia. Athletes with CAI and elevated kinesiophobia demonstrated longer AP‐TTS and greater AP COP velocity than CAI athletes without kinesiophobia, indicating slower and less efficient stabilization following landing. These results suggest that fear of movement or re‐injury may be associated with a more cautious or less adaptable landing strategy.

Interestingly, reduced ML COP velocity variability was observed in the CAI with kinesiophobia group. This pattern may reflect a more constrained postural control strategy, potentially adopted to limit perceived instability or threat during landing [39]. Importantly, these interpretations are based on observable COP behavior rather than inferred neuromuscular or sensory mechanisms, which were not directly measured in the present study.

Kinesiophobia has been associated with altered movement behavior and performance avoidance in athletic populations, particularly following injury [40, 41, 42]. In the context of CAI, fear of re‐injury may influence how athletes interact with the ground during landing, leading to delayed stabilization or altered force‐attenuation strategies [16]. The present findings indicate that kinesiophobia is not merely a psychological construct but is associated with quantifiable changes in dynamic balance performance.

However, it is important to emphasize that while kinesiophobia may contribute to altered stabilization behavior, the underlying mechanisms—such as changes in muscle activation patterns, sensory integration, or motor planning—were not assessed in this study. Therefore, these mechanisms should be considered hypotheses for future investigation, rather than direct explanations of the observed results.

### 4.3 Clinical and Rehabilitation Implications

From a clinical perspective, the results highlight the importance of assessing kinesiophobia in athletes with CAI. Standard rehabilitation programs often focus on strength, proprioception, and neuromuscular control, but may overlook psychological factors that influence movement behavior during high‐demand tasks such as jump landing. The observed association between kinesiophobia and delayed stabilization suggests that integrating psychologically informed rehabilitation strategies, such as graded exposure or confidence‐based landing training, may be beneficial for athletes with CAI who report fear of movement or re‐injury.

Furthermore, COP and TTS measures during dynamic tasks may provide useful objective indicators for identifying athletes who demonstrate maladaptive landing strategies and may be at increased risk for persistent instability or delayed return to sport.

### 4.4 Limitations

Several limitations should be acknowledged. First, this study assessed postural control using force‐plate measures only; joint kinematics, muscle activation (EMG), and sensory manipulation (e.g., visual conditions) were not evaluated. As a result, mechanistic explanations related to neuromuscular or sensory processes cannot be confirmed. Second, the cross‐sectional design precludes causal inference regarding the relationship between kinesiophobia and altered landing stability. Third, the study population consisted of young athletes, which may limit generalizability to non‐athletic or older populations.

### 4.5 Future Directions

Future studies should incorporate longitudinal or interventional designs to determine whether reducing kinesiophobia leads to improvements in landing stability and postural control in athletes with CAI. The inclusion of kinematic analysis, EMG, and sensory manipulation would help clarify the mechanisms underlying the observed COP and TTS differences. Additionally, examining the relationship between kinesiophobia, landing stability, and return‐to‐sport outcomes may provide further clinically meaningful insights.

## 5 Conclusion

In conclusion, athletes with chronic ankle instability and kinesiophobia demonstrate altered landing stabilization characterized by prolonged AP time to stabilization and modified COP behavior. These findings suggest that kinesiophobia is associated with measurable deficits in dynamic postural control during jump landing, beyond the effects of CAI alone. Addressing both physical and psychological factors may be important for optimizing rehabilitation and return‐to‐sport outcomes in this population.

## Author Contributions

**Sara Jafari:** conceptualization, methodology, investigation, data curation. **Sedigheh Sadat Naimi:** conceptualization, methodology. **Zahra Ebrahimabdi:** conceptualization, methodology, formal analysis, writing – review and editing. **Marzieh Mortezanejad:** formal analysis, writing – review and editing. **Aakary Kachoosangy Reihaneh:** writing – original draft **Shanbehzadeh Sanaz:** writing – original draft.

## Funding

The authors have nothing to report.

## Ethics Statement

This study was approved by the Ethics Committee of Shahid Beheshti University of Medical Sciences and Health Services with the ID: IR. SBMU. REC.1401.152. The participants signed the consent form before participating in the study. The authors declare that the manuscript is prepared in personal capacity. This study was done based on the personal capacity of the authors. There are more than 50 universities of medical sciences in Iran, and this study was conducted in Two of them called “the Shahid Beheshti University of Medical Sciences and Health Service”. These universities are not in the list of sanctioned scientific centers. Our activity is only scientific, educational and research. All the authors work only in the university as teacher and researcher and the intended study is the result of their personal capacity. The authors did not conduct this study as ordered by the government or as representatives of the sanctioned government.

## Conflicts of Interest

The authors declare no conflicts of interest.

## Transparency Statement

The lead author Zahra Ebrahimabadi affirms that this manuscript is an honest, accurate, and transparent account of the study being reported; that no important aspects of the study have been omitted; and that any discrepancies from the study as planned (and, if relevant, registered) have been explained.

## Data Availability

Data is available from the corresponding author upon reasonable request.
